# Tunable Multiband Halide Perovskite Tandem Photodetectors with Switchable Response

**DOI:** 10.1021/acsphotonics.2c01328

**Published:** 2022-11-18

**Authors:** Oliver D. I. Moseley, Bart Roose, Szymon J. Zelewski, Simon Kahmann, Krishanu Dey, Samuel D. Stranks

**Affiliations:** † Cavendish Laboratory, University of Cambridge, JJ Thomson Avenue, Cambridge CB3 0HE, U.K.; ‡ Department of Chemical Engineering & Biotechnology, University of Cambridge, Philippa Fawcett Drive, Cambridge CB3 0AS, U.K.; ⊥ Department of Semiconductor Materials Engineering, Faculty of Fundamental Problems of Technology, Wrocław University of Science and Technology, Wybrzeże Wyspiańskiego 27, 50-370 Wrocław, Poland

**Keywords:** perovskite photodetector, multiband, switchable response, narrowband, optical communication, encryption

## Abstract

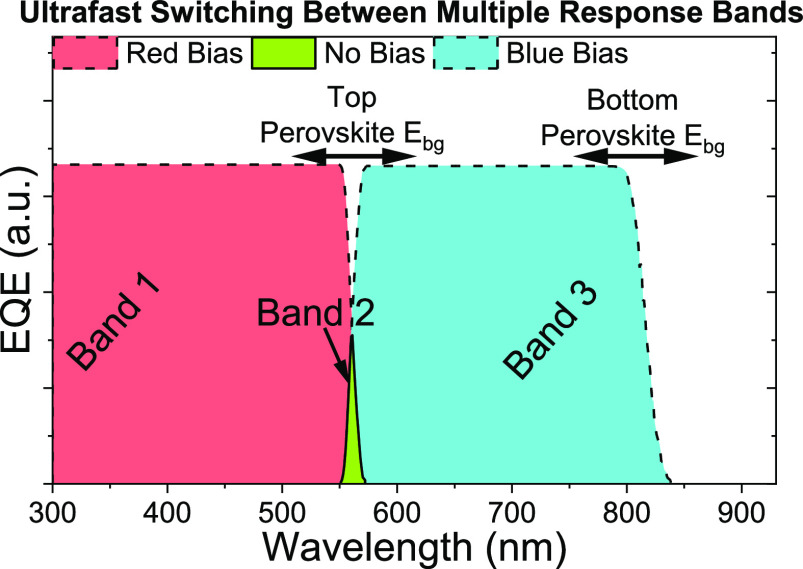

Photodetectors with multiple spectral response bands have shown promise to improve imaging and communications through the switchable detection of different photon energies. However, demonstrations to date have been limited to only two bands and lack capability for fast switching in situ. Here, we exploit the band gap tunability and capability of all-perovskite tandem solar cells to demonstrate a new device concept realizing four spectral bands of response from a single multijunction device, with fast, optically controlled switching between the bands. The response to monochromatic light is highly selective and narrowband without the need for additional filters and switches to broader response bands on applying bias light. Sensitive photodetection above 6 × 10^11^ Jones is demonstrated in all modes, with rapid switching response times of <250 ns. We demonstrate proof of principle on how the manipulation of the modular multiband detector response through light conditions enables diverse applications in optical communications with secure encryption.

## Introduction

Metal halide perovskites have emerged as remarkably defect-tolerant solution-processed semiconductors. Strong direct band gap absorption and excellent charge transport properties^1^ have enabled high-performance perovskite solar cells, exceeding 25% power conversion efficiencies with single junctions and all-perovskite tandems reaching 26.4%,^2^ in just over a decade of development.^3^ Perovskite photodetectors have also rapidly advanced, offering detectivities over 10^13^ Jones^4^ and nanosecond response times,^5−7^ rivaling the performance of commercial photodetectors.^8^ Moreover, compatibility with facile and low-temperature deposition methods, combined with tunable properties through compositional engineering, has opened up a range of novel photodetection functionalities.^9−11^

Whereas most detectors are classified by the spectral selectivity as either narrow or broadband, a third class of devices can act in more than one spectral range. Dual-band detection originally emerged for multicolor IR imaging,^12,13^ requiring expensive and complicated epitaxy with inorganic semiconductors,^14^ thereby restricting applications primarily to military use. Reducing device costs will open up more affordable applications, and recently, solution-processed equivalents using quantum dots,^15^ organics,^16,17^ and perovskites^18−21^ have emerged. These devices allow switchable performance, often by applying bias^22−24^ or illuminating different sides of the device.^25^ However, so far, only two separate response bands are produced from a single device, and importantly, these mechanisms are yet to quantify switching times. Fast switching between detection bands has not been demonstrated and would enable optical gating of detection bands, while more bands of detection would enable greater color resolution, opening up a wider range of applications.

Here, we demonstrate a multiband perovskite photodetector using a two-terminal tandem photodetector (TPD), displaying rapid and selective switching between bands using bias light sources. We exploit the unique properties of a TPD when utilizing a wider band gap perovskite responsible for the color selectivity in the top subcell, combined with a narrower band gap perovskite layer in the bottom subcell. In these devices, monochromatic excitation results in a narrowband response due to the series connection imposed by the two-terminal design and the current matching requirements of both subcells. However, in the presence of white incident light, or by applying specific monochromatic bias light, the behavior shifts to broader response bands, demonstrating a unique ability to optically gate the response to certain colors of light. Four detection modes are offered in total: narrowband, top subcell broadband, bottom subcell broadband, and the ultra-broadband response of both subcells combined, and the spectral positions of these bands can be controlled through compositional engineering of the perovskite absorber layers. We propose a model to explain the device response and use this understanding to optimize the performance in the narrowband mode, leading to full-width-half-maximums (FWHMs) of 16 nm, detectivities over 6.0 × 10^11^ Jones, and <250 ns response time, with a peak response that is tuned from 545 to 800 nm. The broadband modes are also sensitive with detectivities all exceeding 7.0 × 10^11^ Jones. The unique behavior of the TPD device opens up a wide range of new applications and we propose that the ultrafast switching will allow for the selective detection or rejection of certain incident light energies, useful, for example, in microscopy. The novelty of having many response bands could also bring advantages to secure optical communications, and we show that combining pulses of monochromatic light with different wavelengths allows for encryption of information through demonstration of encoding and decoding of a three-color light series.

## Results and Discussion

### Working Mechanism

**Figure 1:**
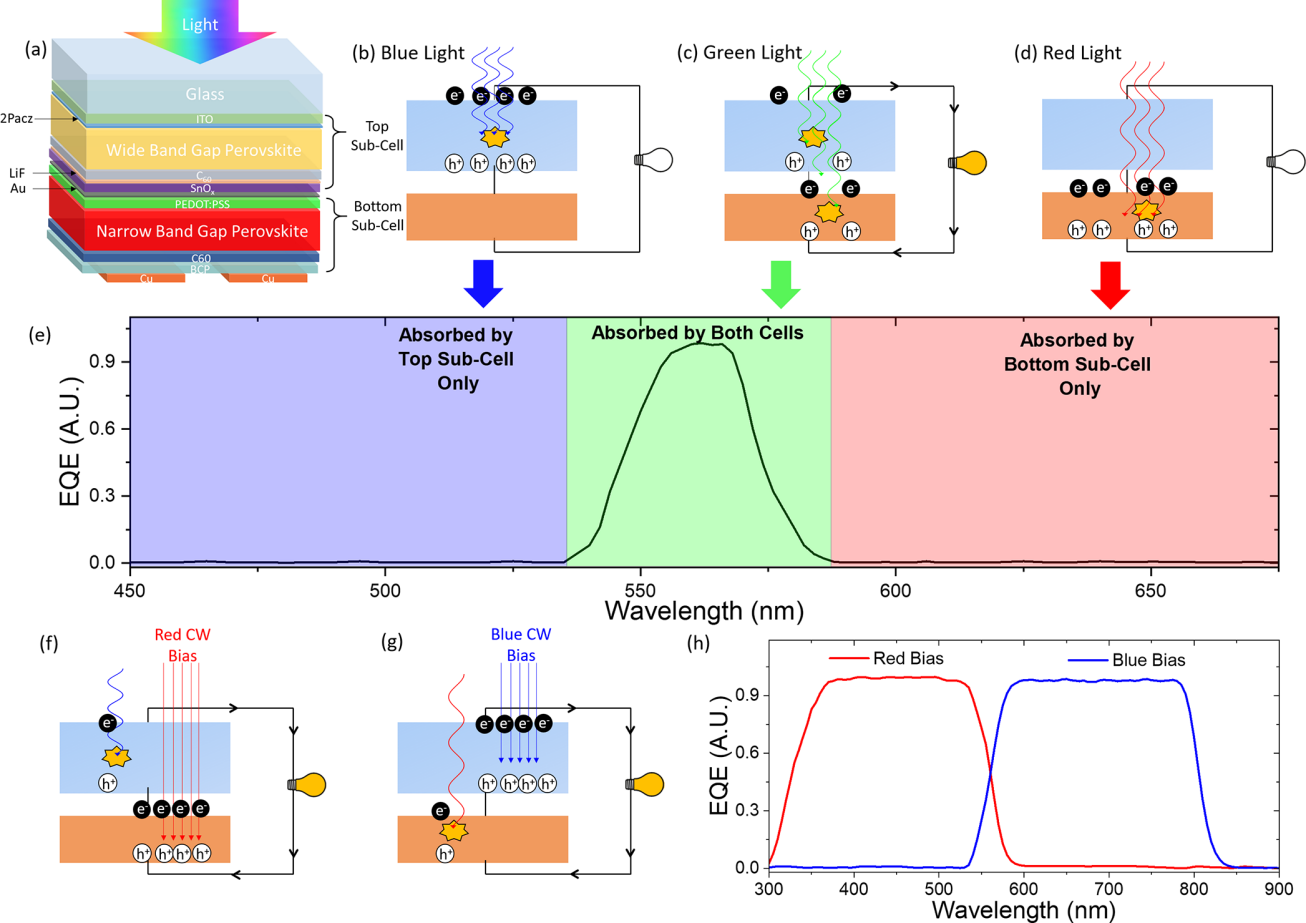
Device structure and detection mechanism. (a) Schematic diagram of the device structure of the two-terminal tandem detector. Schematics of absorption in both subcells to (b) blue, (c) green, and (d) red light, with the corresponding EQE denoted in panel (e). The application of red and blue continuous wave (CW) bias light is shown in panels (f) and (g) respectively, with the corresponding EQE (h) using an optically chopped probe light. Continuous wave bias light saturates one subcell, allowing the chopped light to probe the response of the other, current-limiting subcell. Switching between the narrowband response (e) and two broadband modes (h) is controllable with bias light color.

The TPD has the device architecture shown in Figure 1a, akin to two series-connected p–i–n photodiodes or subcells, each with a perovskite absorber. The same device structure is used for tandem solar cells, which exploit the two absorbing junctions to increase the maximum possible conversion efficiencies over single-junction devices.^26^ Here, we have optimized absorption in the perovskite layers to suit multiband photodetection. The band gap of perovskites can be tuned by changing the constituent ions, and the top subcell (the subcell through which light first enters the device) is designed to have a wider band gap than the bottom subcell by varying the bromide–iodide ratio. As a result, higher-energy light is absorbed in the top subcell and lower-energy light in the bottom subcell. The two-terminal structure connects the subcells in series, which limits the current generated by the TPD to the lowest current from the two subcells, and therefore, both subcells need to be absorbing to generate a response from the detector. Under broadband illumination, such as the AM1.5 solar spectrum, both subcells will be absorbing light and generating current. However, under monochromatic excitation, the response will differ depending on the photon energy and is divided into three categories. On ensuring that perovskite thickness exceeds absorption depths for above band gap photons, higher-energy light, *hν* > *E*_BG_^wide^, will be absorbed primarily in the top subcell (Figure 1b). Similarly, light of *E*_BG_^narrow^ < *hν* < *E*_BG_^wide^ will be absorbed solely by the bottom subcell (Figure 1d), and in both cases, no response will be detected, as the output of the TPD will be limited by the nonabsorbing subcell. However, when *hν* is approaching *E*_BG_^wide^, the absorption of the top subcell begins to decrease and an increasing fraction of light is transmitted through this subcell to reach the bottom subcell (Figure 1c). This allows the bottom subcell to also absorb light, and the TPD will generate a photocurrent. The small range of monochromatic photon energies that generate current in both subcells corresponds to a narrow band of photon detection for the complete device (Figure 1e).

A narrowband response is seen when no other light sources are incident on the TPD. However, on applying continuous wave bias light the behavior shifts back to broadband detection. The shift from narrowband to broadband detection in the presence of bias lights prevents the color-selective detection of white light; however, it opens up unique multiband applications. Bias light of specific wavelengths can selectively saturate a single subcell (Figure 1f,g), and the device current will therefore be limited by the nonabsorbing subcell. This generates two more bands of response: the first up to *E*_BG_^wide^ (top subcell broadband) and the second from *E*_BG_^narrow^ to *E*_BG_^wide^ (bottom subcell broadband, Figure 1h). Similarly, two bias lights (or white bias light) can saturate both subcells, allowing a wide broadband response up to *E*_BG_^narrow^. The spectral positions of all four of these bands, including the narrowband peak, can be tuned with the perovskite band gap, and exploiting the use of bias light allows the response of the device to be selectively switched between the modes.

### Modeling Device Performance

**Figure 2:**
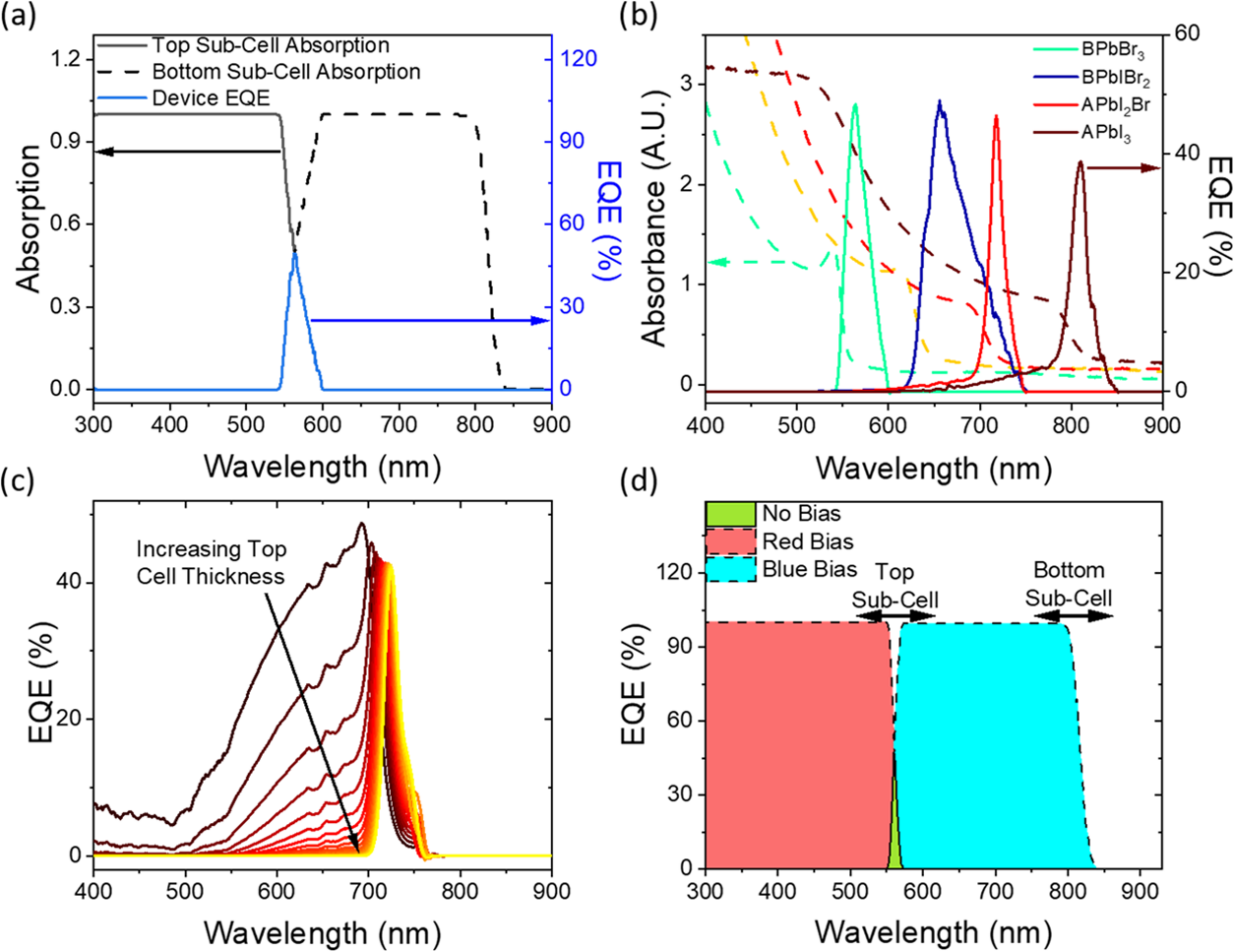
Modeled absorption and device response with different perovskite absorber compositions. (a) Absorption (black lines) in the wide band gap (BPbBr_3_, solid line) top subcell and narrow band gap bottom subcell (APbI_3_, dashed line) in the TPD structure, and the corresponding EQE (blue line) from the full TPD device. A and B refers to Cs_0.05_FA_0.79_MA_0.16_ and FA_0.83_MA_0.17_, respectively. (b) Absorption data for different perovskite thin films (dashed lines), with the modeled EQE (solid lines) when used as a 500 nm top subcell absorber, combined with a 500 nm APbI_3_ bottom subcell. (c) Increasing thickness of an APbI_2_Br top subcell from 100 to 1000 nm (50 nm steps), combined with an APbI_3_ bottom (1000 nm thick) subcell. (d) TPD device EQE with no bias light (green), and red and blue bias lights, highlighting the tunability of all three bands with absorber band gap.

We demonstrate the optical concepts of the TPD behavior to a monochromatic light probe by modeling the absorption and current generation in each subcell (see the Supporting Information, Note 1 for details), and Figure 2a confirms that the narrowband response occurs when both subcells are absorbing the incident light. Inputting absorption data from perovskite thin films of varying composition (by varying the iodide to bromide ratio) into the model demonstrates the impact of band gap and thickness on photodetection (XRD data of corresponding films in Figure S1). Figure 2b shows that the wavelength of the narrowband response is dictated by the top subcell with the wider band gap. Furthermore, the selectivity of this peak is optimized with the top subcell thickness (Figure 2c): increasing thickness reduces the transmission of photons above the energy of the narrowband peak, decreasing the size of any side shoulders and the FWHM. While increasing the wide band gap thickness should continually improve selectivity, the sensitivity will eventually begin to drop once the required charge-carrier transport length exceeds diffusion lengths of carriers, though we note that this is not included in the model due to the assumption of 100% internal quantum efficiency.

A smaller band gap offset between the subcells requires more top subcell thickness optimization to produce a highly selective narrowband response. The extreme case of using the same absorber for both cells is possible, and would enable narrowband detection of white light, but is prone to shoulders on the narrowband response and sensitivity losses (Figure S2). The role of the bottom subcell is to absorb the light transmitted through the top subcell and thus requires a smaller band gap. The thickness of the bottom subcell is less important, so long as it can sufficiently harvest above-band gap photons, while its band gap does not affect the spectral position of the narrowband peak (Figure S2).

The response after the introduction of bias lights is shown in Figure 2d, where saturating a subcell with bias light allows the response to switch to either of two broadband detection bands. The band gaps of both subcells dictate the onsets of these bands, demonstrating that the position of these bands is also tunable. The concept thus generalizes to other semiconductor materials that maintain these requirements, including other absorber materials used for multijunction cells, and we herein continue to demonstrate the concept with halide perovskites.

### Photodetector Performance

Following the results of the model, proof-of-concept devices were fabricated consisting of a Cs_0.05_FA_0.79_MA_0.16_PbI_2_Br layer in the top subcell and Cs_0.05_FA_0.79_MA_0.16_PbI_3_ in the bottom subcell. This was used as the archetypal device for the remainder of the study. Scanning electron microscopy was performed to ensure pinhole-free films were formed (Figure S3), where pinholes would otherwise reduce absorption, allowing shorter wavelength light to pass through the top subcell and to reduce selectivity.

We then assessed the detection of monochromatic light, and initial EQEs displayed the expected narrowband response peak centered at 705 nm. However, they also contained side peaks in the blue (335–450 nm) and NIR (740–825 nm) regions of the spectra (Figure S4). These side peaks represent photon energies where only one subcell will be absorbing, and therefore, the response from the overall device will be coming from the current-limiting nonabsorbing subcell. Reducing the current produced in the nonabsorbing subcell improves selectivity, and studying the origins of this leakage current allowed narrowband performance to be optimized through probe light intensity, probe light frequency, and applied bias, as summarized below (see the Supporting Information, Note 2 for full details).

The current from the nonabsorbing cell is comparable to the dark current of a single-junction photodiode, and therefore, reducing the magnitude of this current was possible with externally applied biases. Fixing the external bias at 0 V (as performed in initial, nonselective measurements) and illuminating only one subcell drive the nonabsorbing subcell into reverse bias, a result of the photovoltage generated in the absorbing cell.^27^ More dark current is expected under reverse bias due to charge injection from the contacts,^28^ contributing to larger side peaks. However, applying a positive bias across the entire device can counteract the photovoltage generated in the absorbing cell and decrease the reverse bias applied to the nonabsorbing cell. A forward bias of 0.5 V was found to be optimal for improving selectivity and used as a standard in measurements (Figure S6).

Probe light intensity impacts the relative magnitude of the photoresponse compared to the leakage current, and as a result, side peaks are more significant at low incident intensities, where this leakage current is of a similar size to the photocurrent. Therefore, greater selectivity is achieved at higher probe light intensities (Figure S4). Studying the intensity dependence of the side peaks using 405 and 800 nm light displays a clear threshold where the current-limiting cell becomes the absorbing subcell, after which point the selectivity increases rapidly with intensity (Figure S4) and both the blue and NIR side peaks drop below 1% EQE above illumination intensities of 2 mW cm^−2^.

**Figure 3:**
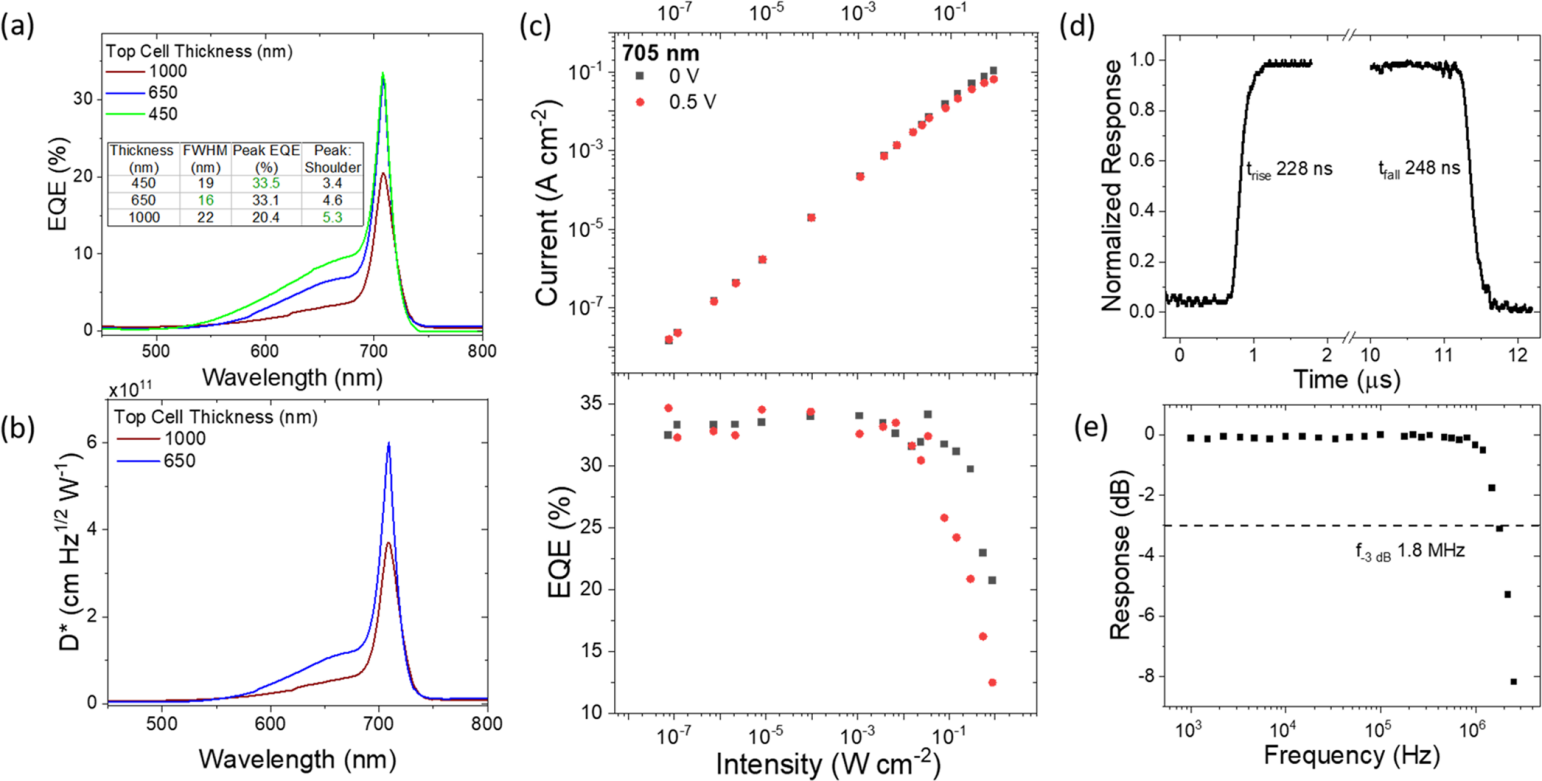
Narrowband photodetector characterization. (a, b) Effect of top subcell perovskite (Cs_0.05_FA_0.79_MA_0.16_PbI_2_Br) thickness on selectivity and sensitivity, panel (a) showing EQE and panel (b) the corresponding detectivity. The detectivity was calculated using the noise current, shown in Figure S9. The highest performing figures of merit are highlighted in green text within the inset table. (c) Linear dynamic range of the narrowband peak (705 nm) represented as the increase in current density (top) and corresponding EQE (bottom) as light intensity is increased from 10^–7^ to 10^–1^ W cm^–2^. Note, the lowest intensity and current were set by the resolution limitations of the measurement setup. (d) Transient response to 670 nm square-wave pulsed light for the TPD. Rise and fall times were taken from the time for the signal to change from 10 to 90% of the peak response. (e) Corresponding cutoff frequency for the same device. The limiting response speed of the experimental instrumentation is included in Figure S10.

Following the understanding of measurement conditions on narrowband performance, the device was optimized to improve selectivity. As shown in the model (Figure 2c), thick perovskite films in the top subcell are a requirement for a selective response. The thickness of the top subcell perovskite was varied through precursor concentration, and the effect on EQE is shown in Figure 3a. Increasing thickness of the top cell absorber reduces the shoulder of the narrowband peak by increasing the absorption of these wavelengths in this top subcell. The same trend is seen for a smaller band gap top subcell perovskite (Figure S7). While increasing thickness will further improve selectivity, at thicknesses of 1000 nm the sensitivity has decreased, due to finite charge diffusion lengths leading to incomplete collection of charge carriers over such large distances. Therefore, a trade-off exists, which can be tuned depending on the requirements of the device. EQEs of 33.5% with a 19 nm FWHM at 705 nm were achieved, and this value increases to 36.4% EQE and 21 nm FWHM at 755 nm for the CsFAMAPbI_2.5_Br_0.5_ top subcell device (Figure S8). This represents the highest sensitivity for a filterless narrowband photodetector at low applied bias.

The photodetection performance of the optimized TPD was then analyzed. The frequency dependence of the noise current was recorded by applying a fast Fourier transform to the time dependence of the dark current (Figure S9). The noise shows little bias dependence between 0 and 0.5 V and gives values of 113 fA/Hz^1/2^ at 20 Hz. Combined with the responsivity values of 0.19 AW^–1^ at 705 nm, this corresponds to peak detectivities of over 6.0 × 10^11^ Jones (Figure 3b), the highest reported value for narrowband perovskite photodetectors to date. At the narrowband wavelength of 705 nm, linear dynamic ranges (LDRs) are observed to be over 113 and 126 dB at 0.5 and 0 V, respectively, limited by the lower limits of the measurement setup (Figure 3c). This matches the best perovskite narrowband performance from charge collection narrowing mechanisms,^29−31^ and is only bettered by broadband detectors, which can exceed 200 dB.^4,32^ The response speed is another important feature of photodetection, and the rise/fall times to pulsed light were measured to be 228/248 ns, respectively (Figure 3d), with a −3 dB cutoff frequency of 1.8 MHz (Figure 3e). These results compare favorably to the response speeds offered by the charge collection narrowing mechanism,^25,31,33^ in that case limited by the low transit times of charges across the thick perovskite layers needed in those devices. The mechanism of switching between modes of TPD operation is controlled by bias lights, and so fast detection times correspond to fast switching times between modes, a feature not seen in previous multiband detection. Further improvements in response speed may be possible by reducing the active pixel area to reduce the RC constant of the device.

**Figure 4:**
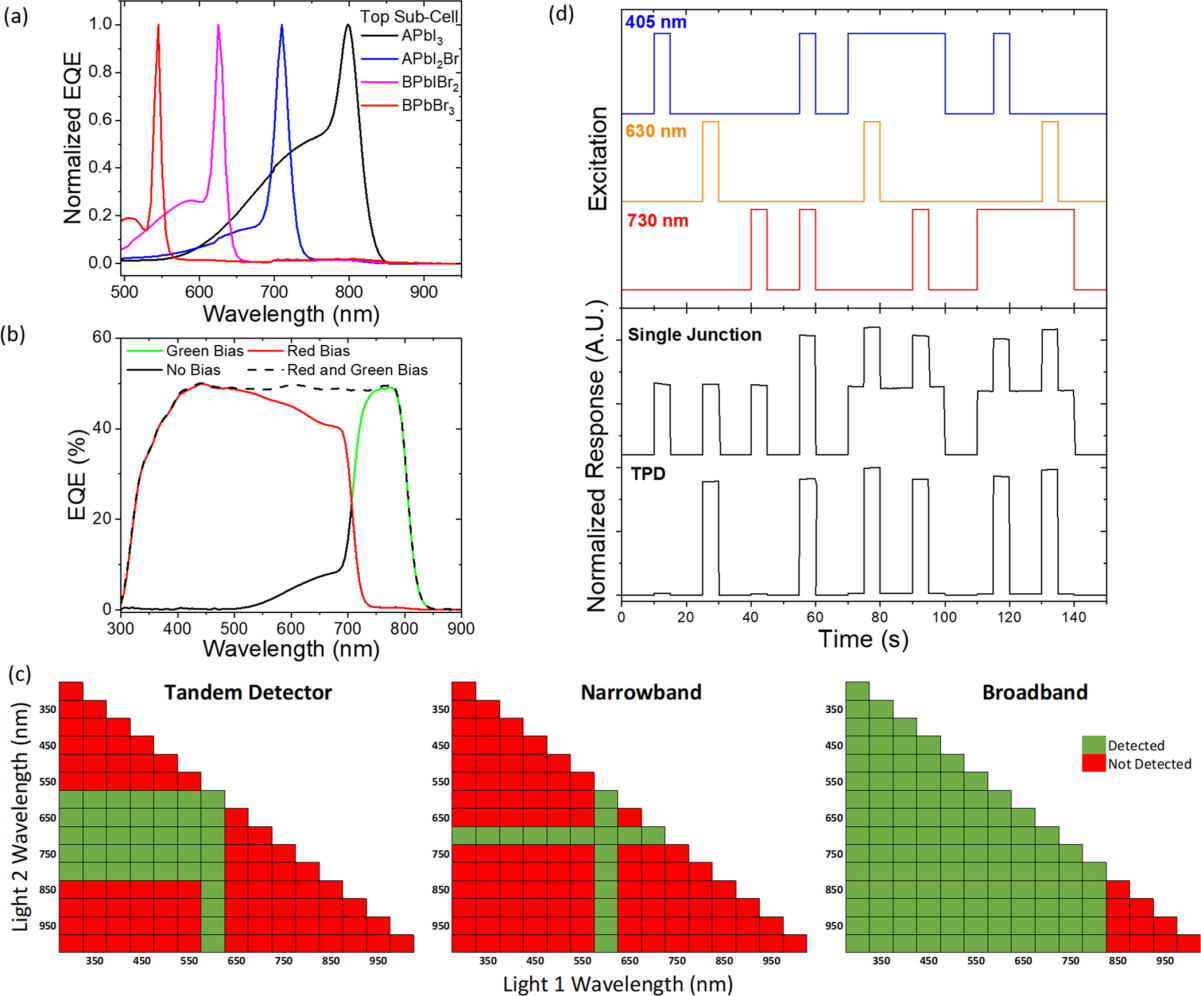
Tunable and switchable multiband detection. (a) Tuning the narrowband response peak with perovskite halide ratios of the top subcell (note the bottom subcell was consistently APbI_3_. a and b refer to Cs_0.05_FA_0.79_MA_0.16_ and FA_0.83_MA_0.17_, respectively). (b) All possible bands of response, depending on bias light color. (c) Matrix of detection for a two-color dual light excitation for three different perovskite detectors. The narrowband detector response is centered at 600 nm, and the single-junction broadband detector has a band gap of ∼800 nm. The symmetry of the response means only half the matrix is shown. The threshold for detection was set so that only one of the two pulses require absorbing in a single-junction detector for a signal to be registered. (d) Response of a perovskite TPD and single-junction photodetector (∼760 nm band gap) to a pulse train of three light sources.

Through compositional tuning of the top subcell perovskite halide site with bromide and iodide, we also demonstrate TPDs with narrowband response peaks between 545 and 800 nm (Figure 4a). We note that the perovskite thickness was kept constant at ∼450 nm, and increased selectivity would be afforded with thicker depositions. Tuning the top subcell perovskite band gap allows a narrowband response across most of the visible and into the NIR spectral region using the TPD device structure. Similarly, this band gap also controls the switchover wavelength between the top and bottom subcell broadband responses. As predicted, the band gap of the bottom subcell has negligible influence on the narrowband peak wavelength (Figure S11), instead controlling the bottom subcell broadband spectral position.

### Multiband Detection

Measuring TPDs in the dark gives a narrowband response, but the energy bandwidth of photodetection can be switched by applying bias light of different colors, demonstrating a multiband response. Saturating one or both subcells with charge carriers by selective continuous wave illumination will change the response of the device to a pulsed probe light. The response of a TPD when the top and bottom subcells are saturated with green or red bias light, respectively, is shown in Figure 4b.

Using the measured performance of the TPD, we highlight the unique response of a TPD versus a narrowband photodetector and a broadband detector of similar band gap to the bottom subcell with a matrix of response to two monochromatic light sources (Figure 4c). The detection of a 400 nm probe source by the TPD will depend on the application of a second light at 600–800 nm. Only by sending both pulses simultaneously will the TPD detect light, whereas individual pulses of light will be (electrically) rejected. In contrast, broadband detectors will detect either one or both of the individual pulses, depending on the semiconductor band gap. Combined with fast switching at above MHz bandwidths, this allows control of the detection of certain colors to be switched in situ and demonstrates a unique mechanism of gating the response, which may have applications in selective microscopy.

We also propose that the unique multiple bands of response would be advantageous for encrypting optical information. To demonstrate this concept, we use this method to consider coded optical pulses, whose deciphering will depend on the detector. Figure 4d shows a pulse train of three different light sources: 405 and 730 nm, to selectively excite the top and bottom subcells, respectively, and 630 nm which corresponds to the peak narrowband response of the TPD device (details in Figure S12 and the Methods section). The response of the TPD device is compared to a single-junction perovskite photodiode, and only when either the 630 nm or both the 405 and the 730 nm probes are sent simultaneously does the TPD afford a significant response. In contrast, the single-junction cell detects each light pulse additively. Therefore, the TPD can uniquely decipher this encrypted optical information, and opens up a number of new applications as a building block in secure optical communications.

## Conclusions

In summary, a tandem photodetector with rapidly switchable and tunable multiband response has been developed and demonstrated with halide perovskite device stacks. A combination of absorption modeling and empirical observations has allowed the mechanism of the behavior to monochromatic and broadband illumination sources to be understood, with the optimum measurement conditions for selective performance uncovered. By controlling measurement conditions, narrowband selectivity is enhanced and, when combined with perovskite thickness tuning, affords narrowband detection exceeding 6.0 × 10^11^ Jones with FWHMs as low as 16 nm. This narrowband peak has been tuned from 545 to 800 nm by varying the iodide to bromide ratio of the perovskite absorbers, with a greater range possible through other compositions and additives including Sn, chloride, and two-dimensional (2D) perovskite systems. Applying bias light to excite both subcells or each subcell individually affords three more detection bands, giving a multiband response that can be selectively controlled. Response speeds to light are ∼250 ns, with bandwidths above 1 MHz, allowing fast optical switching between detection modes. A proof-of-concept pulse train of three different light colors was used to demonstrate the powerful potential for new applications in optical communication.

## Methods

### Fabrication

#### Perovskite Solution Preparation

Cs_0.05_MA_0.16_FA_0.79_PbI*_x_*Br_1–*x*_: Solutions were prepared by dissolving appropriate amounts of formamidinium iodide (FAI, GreatCell Solar), methylammonium iodide (MAI, GreatCell Solar), cesium iodide (CsI, Sigma-Aldrich), lead bromide (PbBr_2_, TCI), and lead iodide (PbI_2_, TCI) in a 4:1 (vol/vol) mixture of *N*,*N*-dimethylformamide (DMF, Sigma-Aldrich) and dimethylsulfoxide (DMSO, Sigma-Aldrich). Perovskite absorber thickness was varied with precursor concentrations, with concentrations of 0.7/0.9/1.1/1.25/1/6/2.0 M producing thicknesses of ∼350/450/550/650/850/1000 nm, respectively. Higher concentrations, and thicknesses, were not possible due to solubility limitations.

#### All-Perovskite Tandem Fabrication

Patterned ITO glass substrates (KINTEC Company) were cleaned using 15 min of sonication in a 2% Hellmanex III (Sigma-Aldrich) solution, followed by 5 min in DI water, 15 min in acetone, and 15 min in isopropanol. The substrates were dried using a nitrogen stream and subjected to a 15 min UV/ozone treatment before being transferred into a nitrogen-filled glovebox. A 1.5 mmol/mL solution of 2PACz in anhydrous ethanol was spin coated at 3000 rpm (5 s ramp) for 30 s, followed by annealing for 10 min at 100 °C. After cooling down to room temperature, Cs_0.05_MA_0.15_FA_0.80_PbI*_x_*Br_1–*x*_ perovskite was deposited onto the substrates by spin coating at 1000 rpm for 10 s (1 s ramp) and 6000 rpm for 20 s (3 s ramp). Anhydrous chlorobenzene was dripped onto the spinning substrate 5 s before the end of the program. The substrates were then annealed for 60 min at 100 °C. The substrates were then transferred to a thermal evaporation system for deposition of 20 nm of C_60_ (Sigma-Aldrich). A 25 nm SnO_2_ interlayer was deposited by atomic layer deposition (Picosun). Tetrakis(dimethylamino)tin(IV) (TDMASn, EpiValence) was used as a precursor and H_2_O as a reactant. The precursor bubbler was heated to 75 °C and the chamber to 100 °C; the reactant vessel was kept at room temperature. The pulsing sequence consisted of a 0.6 s pulse of TDMASn, 10 s purge, 0.1 s pulse of H_2_O, and 10 s purge, resulting in a growth rate of 0.1 nm/cycle. Following ALD, 1 nm of Au was deposited by thermal evaporation. The substrates were removed from the glovebox and a filtered (0.45 μm membrane) 3:1 solution of methanol (Sigma-Aldrich) and PEDOT:PSS (Clevios Heraeus Al 4083) was subsequently spin coated on top of the substrates at 4000 rpm (3.5 s ramp) for 30 s, followed by annealing at 140 °C for 20 min. After removing the substrates from the hot plate, they were immediately transferred to a nitrogen-filled glovebox. Cs_0.05_MA_0.15_FA_0.80_PbI*_x_*Br_1–*x*_ perovskite was deposited onto the substrates by spin coating at 1000 rpm for 10 s (1 s ramp) and 6000 rpm for 20 s (3 s ramp). Anhydrous chlorobenzene was dripped onto the spinning substrate 5 s before the end of the program. The substrates were then annealed for 60 min at 100 °C. After cooling down to room temperature, 20 nm of C_60_, 8 nm of bathocuproine (Sigma-Aldrich), and 120 nm of Cu were deposited by thermal evaporation. Device pixel areas were defined by the evaporation mask to be 0.13 cm^2^.

### Photodetector Characterization

EQEs were measured using a Bentham PVE300 system. A dual xenon short-arc lamp and a quartz halogen lamp were utilized as the light source, with a swingaway mirror set to 750 nm (this is moved if it coincided with a narrowband peak). A 10 × 10 cm Si reference cell was used to calibrate the power of the probe beam. Measurements in AC mode use an optical chopper, with low noise preamplifier and lock-in amplifier as detection electronics. DC measurements had a DC amplifier. External voltages were applied across the device using a Keithley 2450 source measurement unit (SMU). Intensity reductions were made by adding neutral density filters. White bias light was applied with an in-built solar simulator in the system. Red (730 nm, M730L5 Thorlabs) and green (M530L4, Thorlabs) bias lights were applied using LEDs from Thorlabs.

Intensity dependence of the narrowband peak (705 nm) for the LDR measurements were made using a fs laser (InSight DS+ from Spectra-Physics) set to q-CW mode (120 fs pulse, 80 MHz repetition rate is significantly faster than device response times). Power was reduced using neutral density filters from 10^–1^ to 10^–7^ W cm^–2^ and calibrated using a Thorlabs optical power meter with a photodiode sensor. Note, the lowest intensity and current were set by the resolution limitations of the measurement setup. The current was recorded using a Keithley 2450 SMU. The LDR was calculated from the following equation
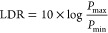
where *P*_max_ and *P*_min_ represent the upper and lower power bounds of the linear response. Noise measurements were made using a Keithley 2450 SMU with different recording rates and a Stanford Research System’s (SRS) SR510 lock-In amplifier, followed by a FFT. Responsivity was calculated using the following equation
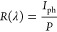
where *I*_ph_ is the device photocurrent and *P* is the incident optical power. The specific detectivity was then calculated using the following equation:
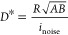
where *A* is the active area, *B* is the bandwidth, *R* is the responsivity, and *i*_noise_ is the noise current.

Transient measurements were performed using a 670 nm fiber-coupled diode laser (iFLEX). The laser beam was passed through an acousto-optic modulator (IntraAction AOM-80, ME-80 driver) to ensure fast excitation and broad frequency range, outputting 100 μW at the first-order diffracted beam. The photodetector current response was recorded with a FEMTO DHPCA-100 transimpedance amplifier combined with an oscilloscope (response time measurement, Tektronix TDS2024C) or a high-speed lock-in amplifier (cutoff frequency measurement, Zurich Instruments HF2LI). The gain of the DHPCA-100 was set to 10^3^ V/A to maximize the bandwidth of the measurement. A Si reference photodiode (Thorlabs FDS010) was used to test the speed limitations of the measurement system. Rise and fall times were taken as the time for the signal to change from 10 to 90% of the peak response.

### Encrypted Communications Demonstration

For the three light source pulse train, a CsFAMAPbIBr_2_//CsFAMAPbI_3_ top and bottom subcell perovskite TPD was used, to allow light sources selectively tuned to each subcell and at the narrowband peak of 630 nm. The EQE of the device is shown in Figure S12. The three light sources were manually switched on and off, and the device response was measured using a Keithley 2450 SMU. The three light sources were a 405 nm CW laser (Photon Etc 405-2W), 630 nm xenon short-arc lamp with a monochromator, and 730 nm LED (M730L5 Thorlabs, combined with 665 nm long pass to minimize any excitation of top subcell). The reference single-junction perovskite photodiode had the structure ITO/2PACz/perovskite/C60/Ag, with a CsFAMAPbI_2.5_Br_0.5_ absorber (∼1.6 eV band gap).

## Data Availability

The data and code that support the findings of this study are available at [http://doi.org/10.17863/CAM.90395] in the University of Cambridge Apollo repository.

## Supporting Information Available

Details on the device modeling, optimization of photodetection performance with measurement conditions and perovskite thickness/band gap, and additional experimental details (PDF)
