# Supplementary material for: Tunable Multiband Halide Perovskite Tandem Photodetectors with Switchable Response
Source: ACS Photonics. 2022 Nov 18;9(12):3958–66. doi: 10.1021/acsphotonics.2c01328 (PMC9782784; doi:10.1021/acsphotonics.2c01328)
Supplement: Supplementary file 1 — ph2c01328_si_001.pdf [file ph2c01328_si_001.pdf]

Supporting Information: Tunable Multiband Halide Perovskite Tandem Photodetectors with Switchable Response

Oliver D. I. Moseley[1#], Bart Roose[1,2#*], Szymon J. Zelewski[1,3], Simon Kahmann[1,2], Krishanu Dey[1], Samuel D. Stranks[1,2*]

1: Cavendish Laboratory, University of Cambridge, JJ Thomson Avenue, Cambridge CB3 0HE, UK.

2: Department of Chemical Engineering & Biotechnology, University of Cambridge, Philippa Fawcett Drive, Cambridge CB3 0AS, UK.

3: Department of Semiconductor Materials Engineering, Faculty of Fundamental Problems of Technology, Wrocław University of Science and Technology, Wybrzeże Wyspiańskiego 27, 50-370 Wrocław, Poland.

* Email br340@cam.ac.uk and sds65@cam.ac.uk.

[#] These authors contributed equally.

Supporting Note 1: Device Modelling

The response to monochromatic light was modelled using the Beer-Lambert absorption of perovskite films with different compositions, to find the distribution of photon absorption between the two sub cells. The absorption data was obtained using a Shimadzu UV-VIS-NIR Spectrophotometer UV-3600 Plus, using a step size of 1 nm and a beam splitter to measure the sample and reference simultaneously.

Note the impact of the other layers in the devices was not accounted for, either for contributions to parasitic absorption or cavity effects. Internal quantum efficiencies of 100% was also assumed, implying that any absorption event would lead to charge carrier extraction in each sub cell. The requirement of current matching was included by taking the limiting current from both of the sub cells as the final output of the device.

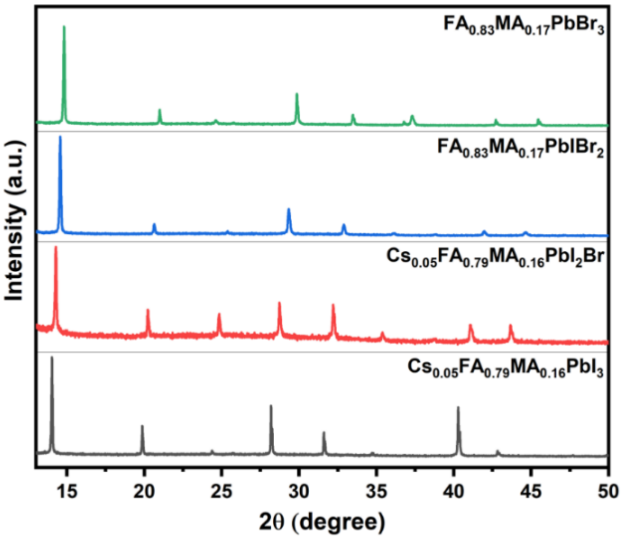

Fig. S1: XRD data on perovskite thin films of varying composition.

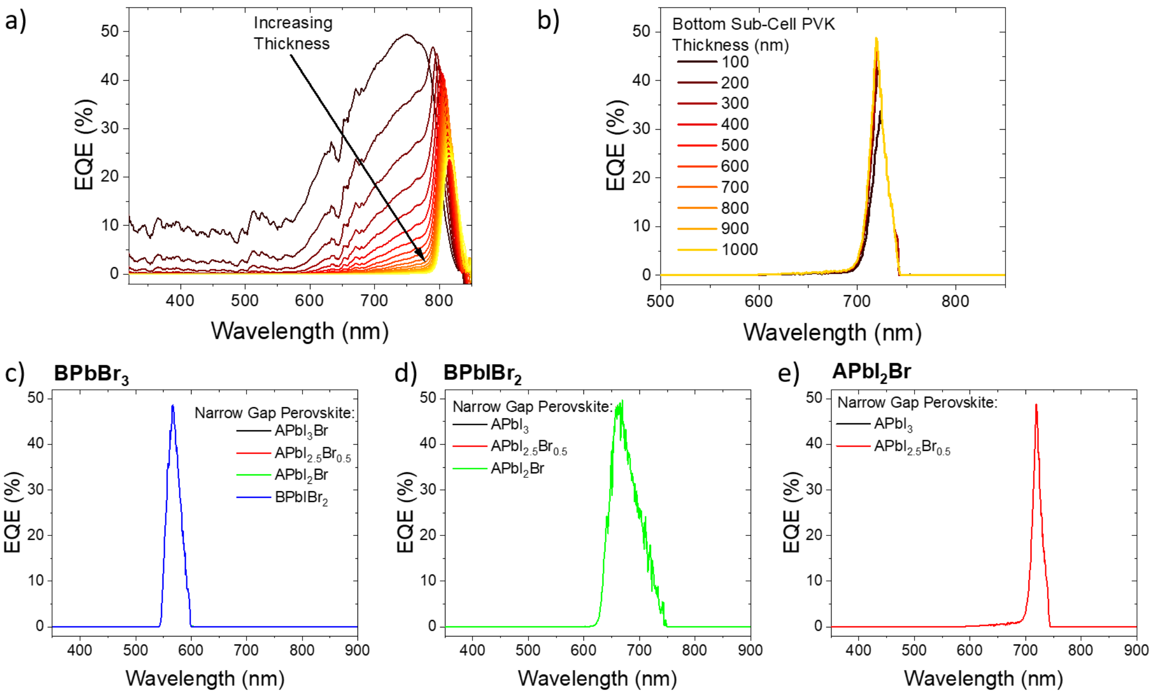

Fig. S2: Device modelling results. *a) Varying the thickness (100 to 1000 nm in 50 nm increments) of the top sub-cell perovskite in a TPD with identical perovskites in both sub-cells ($APbI_3$). (b) Varying the thickness (100 to 1000 nm in 100 nm increments) of the bottom sub-cell perovskite. c-e) Varying the bottom sub-cell perovskite composition whilst keeping the top sub-cell perovskite composition constant for (c) $BPbBr_3$, d) $BPbIBr_2$ and e) $APbI_2Br$ wide band gap perovskite sub-cells. The thicknesses of each perovskite were set to 600 and 1000 nm for the wide and narrow gap cells respectively. The peaks fully overlap, confirming there is no impact on the peak wavelength of narrowband response when varying this narrow band gap perovskite. Note, A and B refers to $Cs_{0.05}FA_{0.79}MA_{0.16}$ and $FA_{0.83}MA_{0.17}$, respectively.*

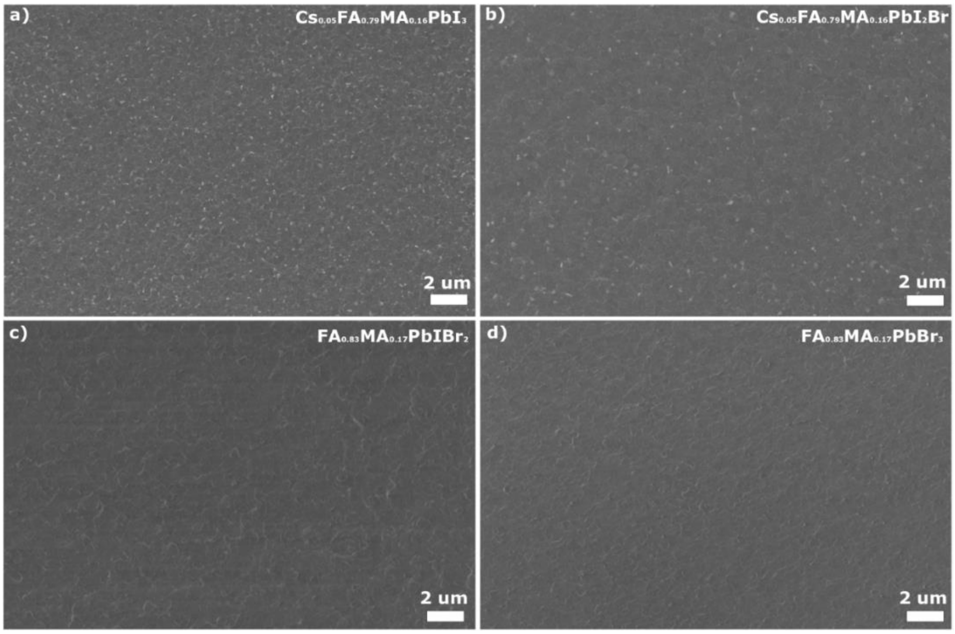

Fig. S3: Scanning electron microscopy (SEM) on perovskite thin films of varying composition. *a) $Cs_{0.05}FA_{0.79}MA_{0.16}PbI_3$ b) $Cs_{0.05}FA_{0.79}MA_{0.16}PbI_2Br$ c) $FA_{0.83}MA_{0.17}PbIBr_2$ d) $FA_{0.83}MA_{0.17}PbBr_3$.*

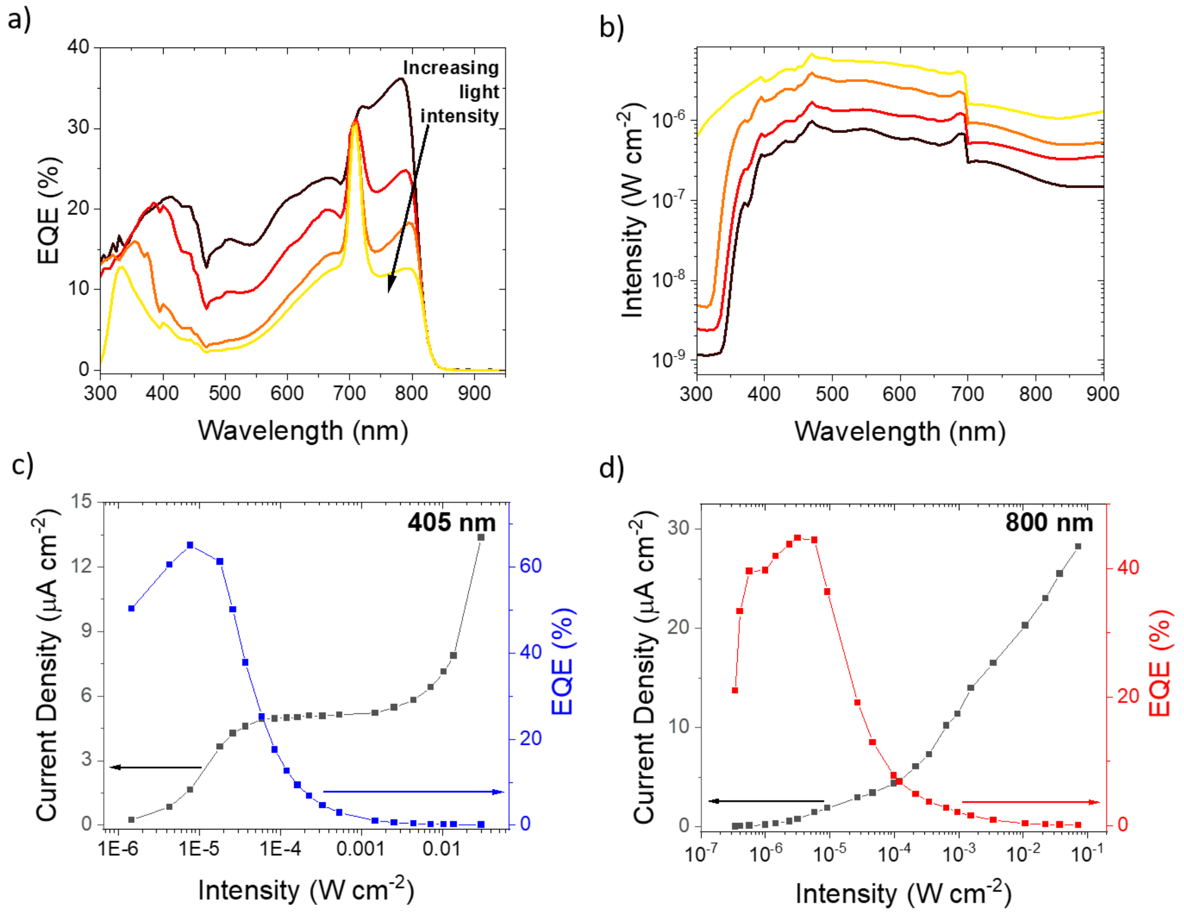

***Fig. S4: Monochromatic probe light intensity in EQE measurements**. a) Intensity dependence on the EQE selectivity. Power was reduced by adding ND filters, with the corresponding probe light intensity shown in b). Below ~350 nm the power falls off significantly, and resultantly the leakage current of the non-absorbing narrow band gap cell is very significant in this region, leading to side peaks in the narrowband response. The light aperture was set to 1.5 x 1.5 mm, smaller than the device pixel area. EQEs were in AC mode at 30 Hz chopper frequency and fixed at 0 V external bias. c) and d) Intensity dependence of side peak current and EQE in the blue (405 nm, c)) and NIR (800 nm, d)).*

Supporting Note 2: Optimising Narrowband Measurement Conditions

1. Intensity Dependence EQE selectivity

External quantum efficiency (EQE) spectra are shown in Fig. S4a with a narrowband peak centred at 705 nm. However, side peaks in the blue (335-450 nm) and NIR (740-825 nm) are also present, hampering the narrowband selectivity. While the EQE of the narrowband peak remains constant when light intensity is reduced, the side peaks become more prominent. In the TPD architecture, the sub-cell that generates the lowest current determines the EQE response. Away from the peak narrowband wavelengths, this will be the non-absorbing sub-cell, which will generate a leakage current similar to the dark current of a single junction photodiode. While the magnitude of these non-absorbing 'dark' or leakage currents are low, they become increasingly significant as the probe beam intensity decreases, generating these side peaks. These effects are exaggerated in our measurement system, due to sharp lamp intensity drop offs below ~430 nm and above ~700 nm (Fig. S4b).

To gain more understanding on this effect, the side peaks were selectively probed over a wider range of intensities using 405 nm and 800 nm monochromatic light, shown in Figures S4c and S4d respectively. For 405 nm irradiation, there are three distinct regimes: an initial increase in current,

followed by a plateau and then a second rise in current. The initial rise represents the top sub-cell being the limiting sub-cell and the current plateaus when the top sub-cell photocurrent exceeds the leakage current in the non-absorbing narrow band gap sub-cell, around 4.9 µA cm^{-2}. The leakage current from the narrow band gap sub-cell is higher than the top sub-cell due to its smaller band gap (generating more thermal carriers) and because the entire TPD cell is held at short circuit. Fixing the bias at 0 V and illuminating only one cell drives the non-absorbing cell into reverse bias, a result of the photovoltage generated in the absorbing cell[1]. More leakage current is expected under reverse bias due to charge injection from the contacts[2]. Reducing leakage current with an external bias, to increase selectivity, is discussed below. Above intensities of 0.2 mW the current begins to increase again, but the EQE continues to decrease. A possible explanation for this third regime is the onset of bimolecular recombination allowing efficient luminescent coupling between the two cells[3] and will be the subject of future investigations. For the side peak at 800 nm the behaviour is less well understood, and the current increases with intensity over the whole measured range. Luminescent coupling is unlikely in this case, as the narrower band gap of the bottom sub-cell would emit photons too low in energy to be absorbed by the top sub-cell. Again, the EQE reaches a peak value (at a similar power to the 405 nm plot), likely representing the switchover of limiting current from the absorbing to the non-absorbing cell, and then continually decreases. The exact mechanism will be the subject of future studies. What is important here is the decrease in sensitivity of this side peak with increasing intensity, allowing higher selectivity of the narrowband peak. Both the blue and NIR side peaks drop below 1% EQE above illumination intensity of 500 µW.

2. Measurement Frequency Dependence

As the side peaks relate to the ‘dark’ or leakage current of the non-absorbing cell, the impact of probe light frequency on selectivity was studied. Previous work has demonstrated transient behaviour of perovskite dark current, especially under reverse bias[4], and so different probe light frequencies will impact this behaviour in different ways. Fig. S5a displays EQEs at different AC light frequencies, and continuous wave illumination with DC detection. As frequency decreases, the side peaks in the blue and NIR decrease, and the selectivity increases. This behaviour is explored in more detail using 405 nm continuous wave excitation and recording the response of the TPD with different measurement intervals as the light source is turned on and off. The response to a single light pulse is shown with different recording rates in Fig. S5b. After switching on the light (at 0 seconds) the current initially peaks before stabilizing, similar to that seen when measuring dark current transients of perovskite photodiodes[4]. A higher sampling rate better resolves the peak current immediately after illumination. This leads to larger signals, and Fig. S5c shows a greater than 100% increase in both current and EQE when measured at 1 versus 97 Hz. This effect only occurs when the leakage current of the non-absorbing cell is current limiting, hence the impact on the side peaks, and consequently the narrowband EQE is unaffected. As a result, continuous illumination and DC measurement displays the best selectivity.

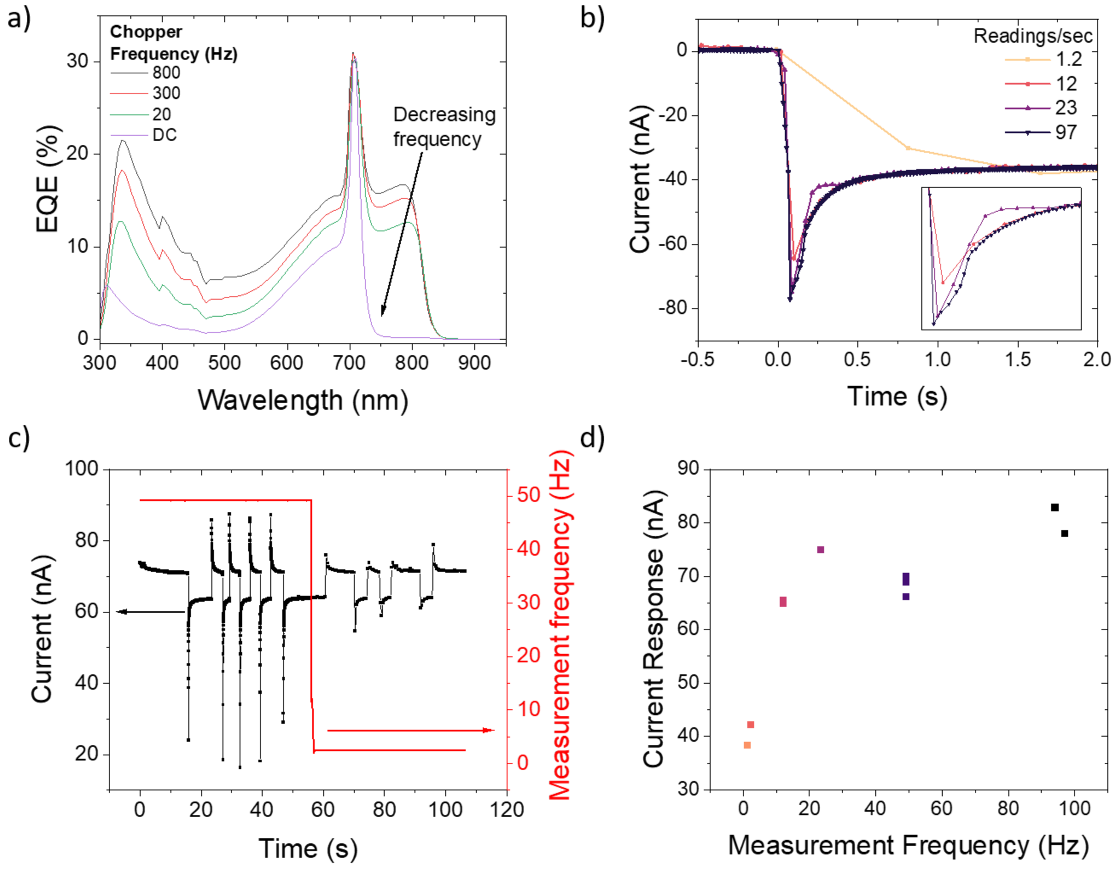

Fig. S5: Impact of measurement frequency on narrowband selectivity. *a) EQE measurements in AC mode at different frequencies, compared to DC mode (violet line). b) Transient response to continuous wave 405 nm light (turned on at 0 seconds) recorded at different measurement rates. The resolution of the initial spike (inset) is improved at higher recording rates. 200 μW light is used to ensure the device is kept in a regime limited by the dark current of the non-absorbing bottom sub-cell. c) Displays the effect on several light on and off values. d) The corresponding difference in current response (I_{on}-I_{off}) when varying the measurement recording rates. Slower recording rates, corresponding to lower chopper frequencies, lead to smaller EQEs.*

3. Applied Bias

As introduced above, the narrowband selectivity is also impacted by the external bias across the TPD, as this dictates the bias applied to the non-absorbing cell, and consequently affects its leakage current. The previous measurements shown have all been carried out at short circuit, and the impact of sequentially increasing external bias is shown in Fig. S6. Applying a positive bias across the entire device, and illuminating with a wavelength that selectively excites one sub-cell, can counteract the photovoltage generated in the absorbing cell, and decrease the reverse bias applied to the non-absorbing cell. Countering the reverse bias reduces the leakage current from injected carriers in this cell, and increases the selectivity of the TPD. This is especially important at low incident light intensities, when photocurrents are low. Around +0.4 V the NIR side peak reaches a minimum, and above +0.6 V the narrowband peak begins to decrease, and thus +0.5 V was selected as the standard.

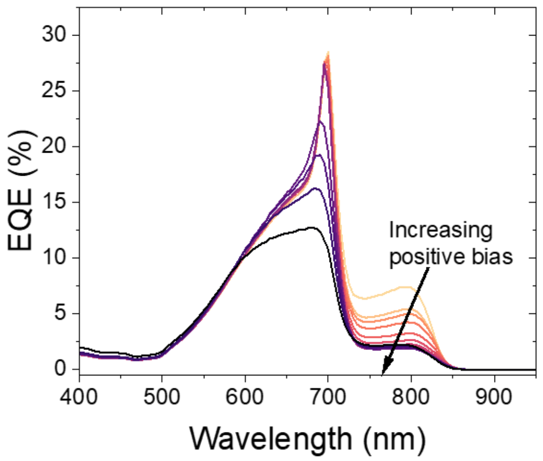

Fig. S6: Impact of applied bias on narrowband selectivity. *Recording EQE with different external biases applied across the whole TPD device, from -0.1 to 1 V (0.1 V step, AC mode, 20 Hz).*

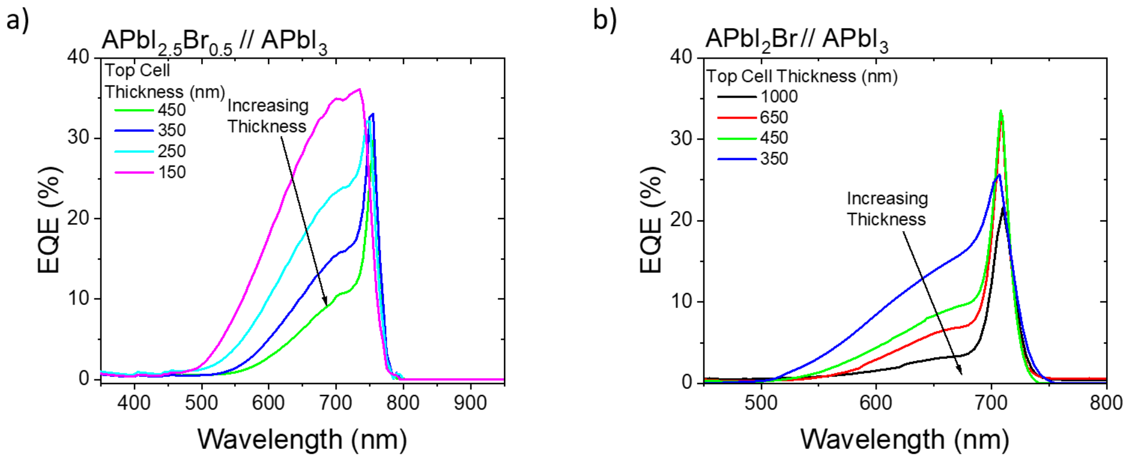

Fig. S7: Improving selectivity by increasing top sub-cell perovskite thickness. *a)* $APbI_{2.5}Br_{0.5}$ *and b)* $APbI_2Br$ *wide band gap perovskite top sub-cells, both combined with a 450 nm* $APbI_3$ *narrow band gap perovskite bottom sub-cell. A refers to* $Cs_{0.05}FA_{0.79}MA_{0.16}$

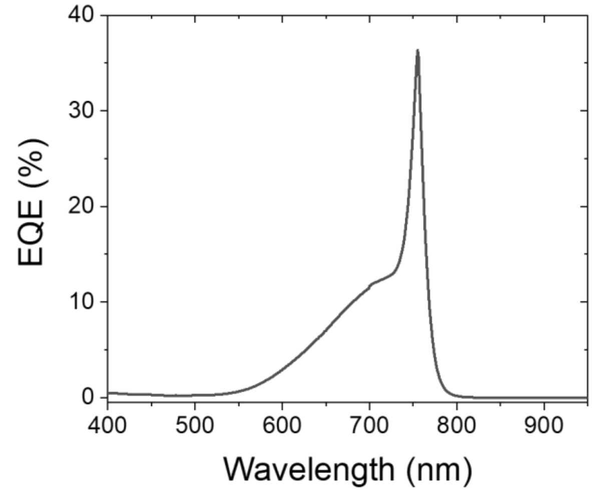

***Fig. S8: Hero device performance for $Cs_{0.05}FA_{0.79}MA_{0.16}PbI_{2.5}Br_{0.5}$ // $Cs_{0.05}FA_{0.79}MA_{0.16}PbI_3$ TPD**. EQE of 36.4% and FWHM of 21 nm.*

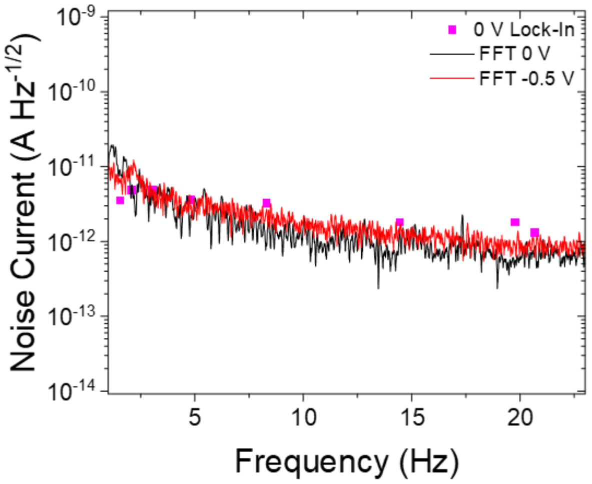

***Fig. S9: Noise spectral density of archetype TPD ($Cs_{0.05}FA_{0.79}MA_{0.16}PbI_2Br$ // $Cs_{0.05}FA_{0.79}MA_{0.16}PbI_3$).** Measurement of current vs time were made using a Keithley 2450 SMU and using a FFT converted to frequency domain. Lock-in measurements were made with a SR510 amplifier set to noise mode.*

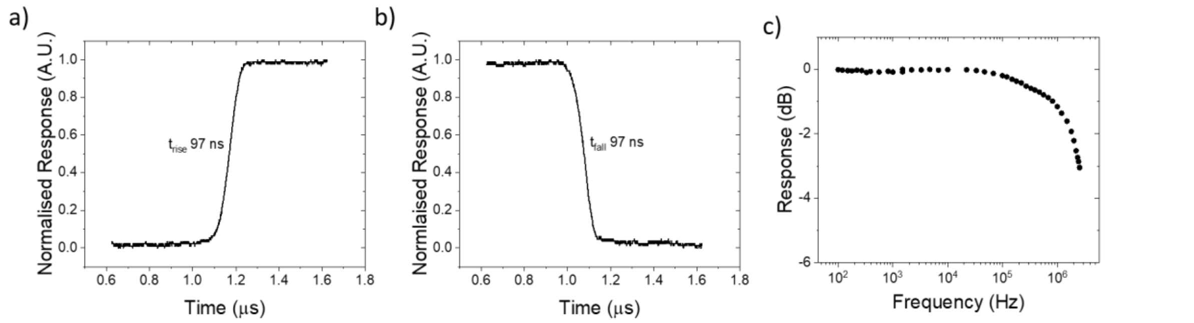

Fig. S10: Response Speed Measurements. *a) and b) Shows response times limits of our rise and fall time measuring instrument using an ultrafast Si photodiode. c) The corresponding cut-off frequency of the Si photodiode, demonstrating a broader frequency response than measured of the tandem photodetector.*

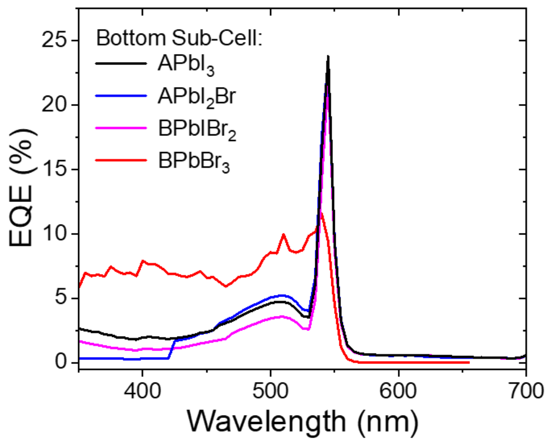

Fig. S11: Narrow band gap bottom sub-cell perovskite composition. *Varying the bottom sub-cell perovskite band gap, with constant $Cs_{0.05}FA_{0.79}MA_{0.16}PbBr_3$ wide band gap perovskite. Both sub-cell thicknesses were not optimised at 450 nm.*

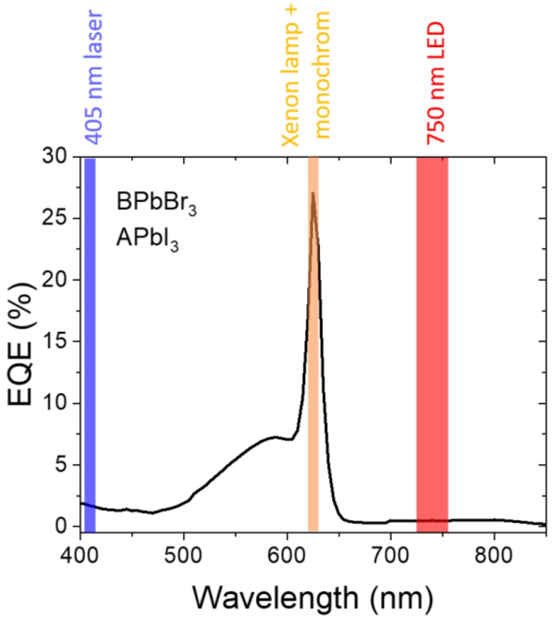

Fig. S12: Pulse train experiment. *Monochromatic light EQE for the $BPbBr_3$ // $APbI_3$ TPD device, with the three light sources used to send light pulses. This top and bottom sub-cell perovskite device was chosen as it allows us to selectively excite each sub cell with the light sources chosen. Note, A and B refers to $Cs_{0.05}FA_{0.79}MA_{0.16}$ and $FA_{0.83}MA_{0.17}$, respectively.*

References

1. Meusel, M. *et al.* Spectral response measurements of monolithic GaInP/Ga(In)As/Ge triple-junction solar cells: Measurement artifacts and their explanation. *Progress in Photovoltaics: Research and Applications* **11**, 499–514 (2003).
2. Tian, W., Zhou, H. & Li, L. Hybrid Organic–Inorganic Perovskite Photodetectors. *Small* **13**, 1702107 (2017).
3. Bowman, A. R. *et al.* Relaxed Current Matching Requirements in Highly Luminescent Perovskite Tandem Solar Cells and Their Fundamental Efficiency Limits. *ACS Energy Lett.* **6**, 612–620 (2021).
4. Bowring, A. R., Bertoluzzi, L., O'Regan, B. C. & McGehee, M. D. Reverse Bias Behavior of Halide Perovskite Solar Cells. *Advanced Energy Materials* **8**, 1702365 (2018).
